# Recurrent metastatic clear cell renal carcinoma with sarcomatoid dedifferentiation treated with surgery and Cabozantinib

**DOI:** 10.18632/oncotarget.27543

**Published:** 2020-05-19

**Authors:** Johnny Boustany, Maher Abdessater, Charbel El Hachem, Ziad El Khoury, Walid El Khoury, Raghid El Khoury

**Affiliations:** ^1^Notre Dame des Secours University Hospital Center (CHUNDS), Byblos City, Lebanon; ^2^Holy Spirit University of Kaslik (USEK), Jounieh, Lebanon

**Keywords:** clear cell renal carcinoma, sarcomatoid dedifferentiation, metastasectomy, cabozantinib

## Abstract

Renal cell carcinoma with sarcomatoid dedifferentiation is an entity of RCC that has undergone an anaplastic transformation with both a carcinomatous and a sarcomatous component. The standard treatment in metastatic patients is immunotherapy.

The aim of this article is to describe our case of metastatic recurrent RCC with sarcomatoid dedifferentiation in a 59 year old male patient treated with nephrectomy and multiple metastasectomies followed by Cabozantinib.

Consecutive PET-CT scans showed no evidence of recurrence, three years after the last metastasectomy, and the patient is having currently a normal life.

Sarcomatoid dedifferentiation remains a poor prognosis factor in RCC. Surgery for metastases followed by Cabozantinib may be a therapeutic option in metastatic young patients. However, a prospective randomized trial would be the best option to validate this approach.

## INTRODUCTION

Renal cell carcinoma (RCC) accounts for 3% of all adult cancers [1].

Clear cell renal carcinoma (CCRC) is a RCC entity that accounts for 66% to 75% of all malignant tumors of the kidney. It occurs between the 5th and 7th decade of life and its most common risk factors are tobacco and obesity [2].

Renal cell carcinoma with sarcomatoid dedifferentiation is an entity of RCC that has undergone an anaplastic transformation with both a carcinomatous and a sarcomatous component. Forty percent of patients with RCC with sarcomatoid dedifferentiation present with high pathologic stage disease (pT3 or pT4), as well, 40% of these patients have metastatic disease at the time of diagnosis [2].

The treatment is not well defined and remains a challenge due to the poor prognosis of this entity.

The aim of this article is to share our experience about a patient who is in complete remission from RCC with sarcomatoid dedifferentiation under cabozantinib after two surgical interventions to excise all the metastases.

## CASE PRESENTATION

A 59 years old non-smoker male patient with no past medical history presented for cough and fever (39°C) since few days.

**Figure 1:**
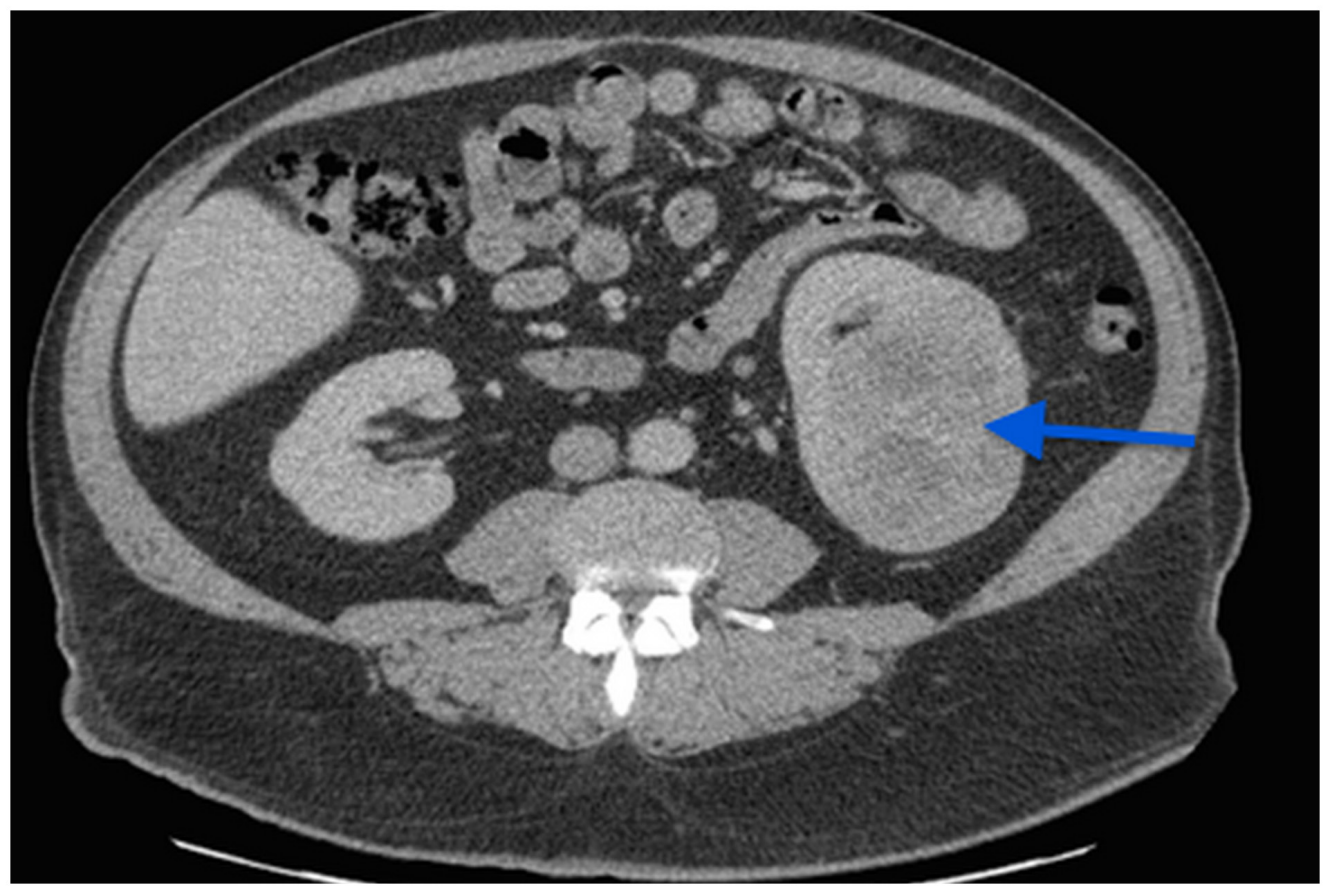
A CT scan image showing a 7 cm left kidney mass.

Chest computed tomography (CT) scan was normal but abdominal CT revealed a 7 cm left kidney mass (Figure 1).

The patient has undergone left laparoscopic radical nephrectomy in our center and was discharged at the second post-operative day without any surgical complication.

Pathology revealed a 7 cm clear-cell renal cell carcinoma of Fuhrman nuclear grade 4, with lympho-vascular permeation and capsular invasion. After the multidisciplinary uro-oncology team meeting, the decision of close follow-up was taken.

Four months later, while the patient was abroad, he had a flu-like syndrome with low grade fever that was treated by paracetamol. No additional investigations were done.

Three weeks later, he consulted his general practitioner for the persistence of the low grade fever and the appearance of anorexia and severe fatigue. Blood tests revealed severe anemia (Hb of 7.9 g/dl), a CRP of 337 mg/l and a normal creatinine level. Urine culture was positive for E. Coli that was treated with ceftriaxone 2 g daily for 4 days without any improvement.

This is why, the patient presented to the ER of another institution, with fever, increased fatigue and distended abdomen. Physical exam showed a 10 cm palpable mass of the right iliac fossa and a subcutaneous 5 cm mass in the left iliac fossa.

Blood test done showed severe anemia (Hb 5.5 mg/dl) and 14000 WBCs per cubic millimeter of blood.

He was admitted to the hospital, and was put under broad spectrum antibiotics.

A thoraco-abdomino-pelvic CT scan showed multiple intra and extra peritoneal masses with suspicion of a small intestine fistula.

Biopsy of the right inguinal mass revealed a clear-cell renal carcinoma with sarcomatoid dedifferentiation. Brain MRI was negative.

Despite antibiotics and multiple blood transfusions, anemia and low grade fever persisted and the multidisciplinary tumor board meeting of that institution has seen that surgery is unreasonable, so the decision was to start an anti-angiogenic treatment despite high risk of infection flair up under such treatment.

**Figure 2:**
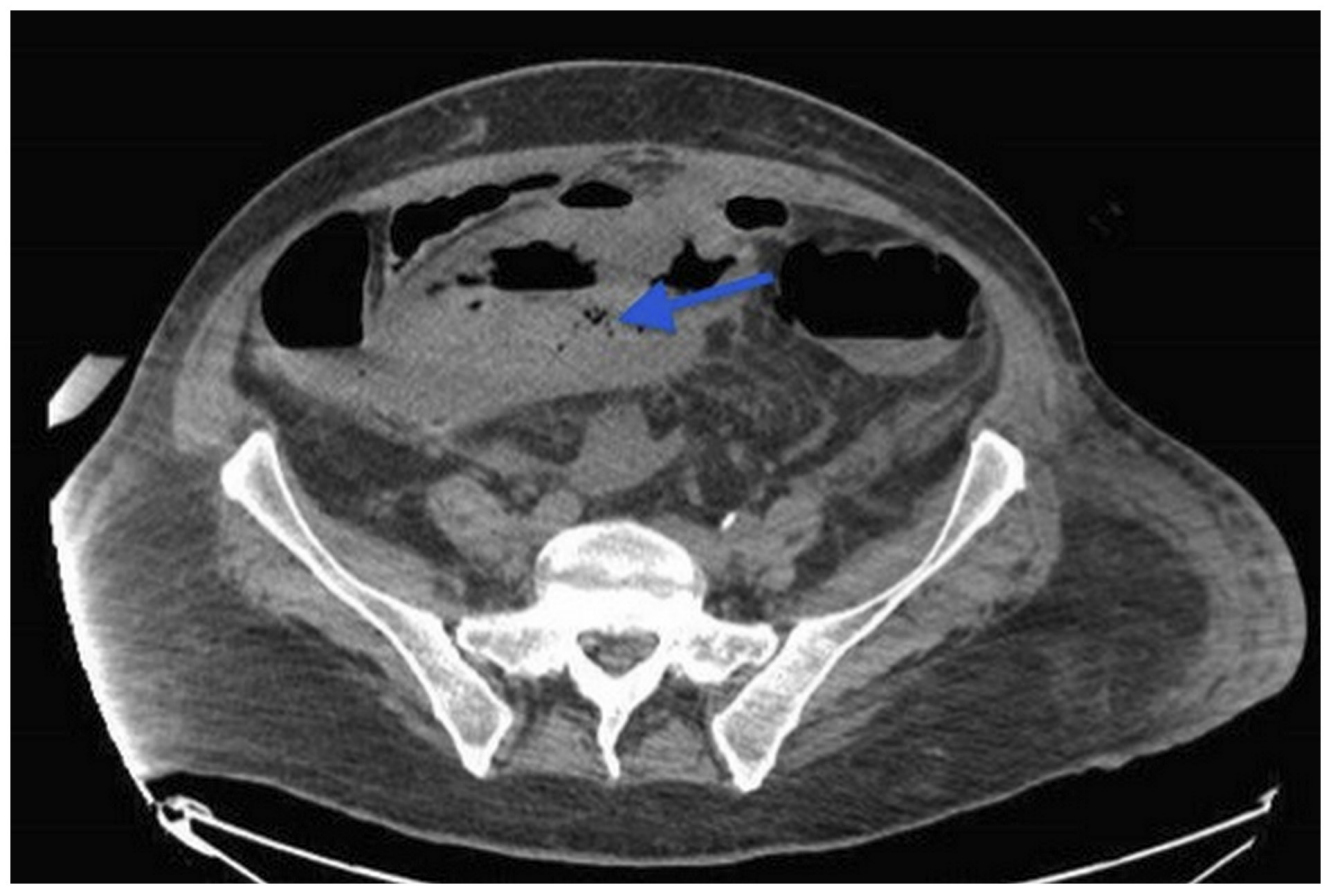
CT scan of the abdomen and pelvis revealed the presence of gas inside the pelvic mass (arrow) due to fistulisation of an intestinal loop with localized peritonitis.

Eight days after the initiation of SUTENT (Sunitinib) 800 mg/day, and in front of the persistent fever and severe abdominal pain, a CT scan of the abdomen and pelvis was done and revealed the presence of gas inside the right inguinal mass due to fistulisation of an intestinal loop with localized peritonitis (Figure 2).

Patient was transferred to ICU, surgical management was refused and percutaneous drainage of the right inguinal collection was done (evacuation of pus).

SUTENT was stopped (despite regression of the anemia) and Meropenem was started. Culture of pus showed E coli (ESBL).

Fever persisted for 4 days and a control CT scan showed a stability of the drained collection but the appearance of a new peritoneal one.

Reevaluation of the case was done by the treating medical team and, due to the altered general status (PS = 4), the surgical management was refused. Thus, palliative care was decided.

However, and responding to the request of his relatives, the patient was transferred back by plane to our institution where he was admitted to the ICU: he was stable but febrile (38.6C), he had oliguria, distended abdomen with multiple palpable masses and a GCS of 13/15.

**Figure 3:**
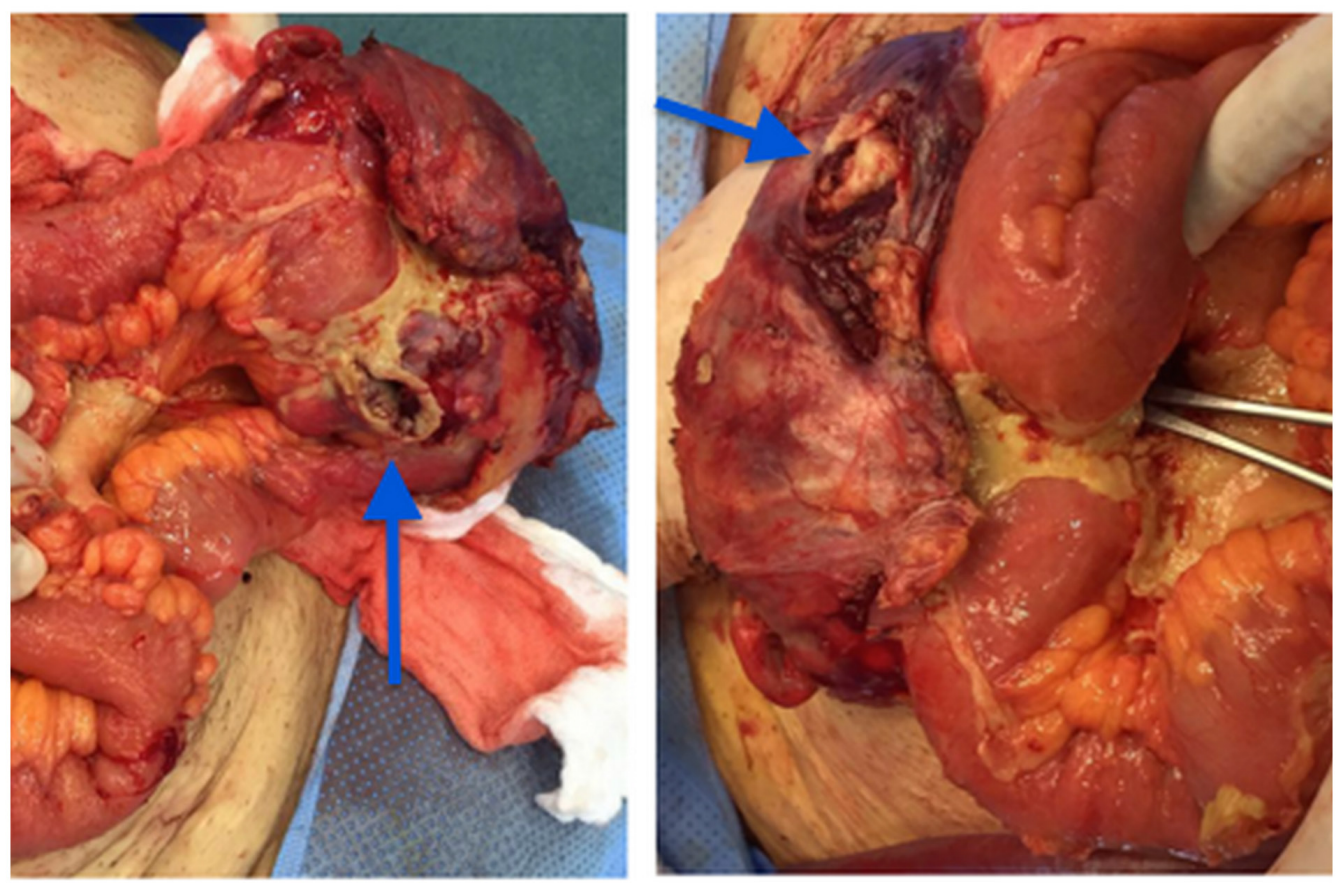
Right inguinal mass in communication with the intestinal loop (arrows).

After the discussion with the patient and his relatives we did a laparotomy for the excision of all the masses and drainage of the collections previously seen on CT. (Figure 3).

The patient stayed in the ICU for 3 days after the surgery. No complications occurred during the recovery phase and he was discharged home one week later.

**Figure 4:**
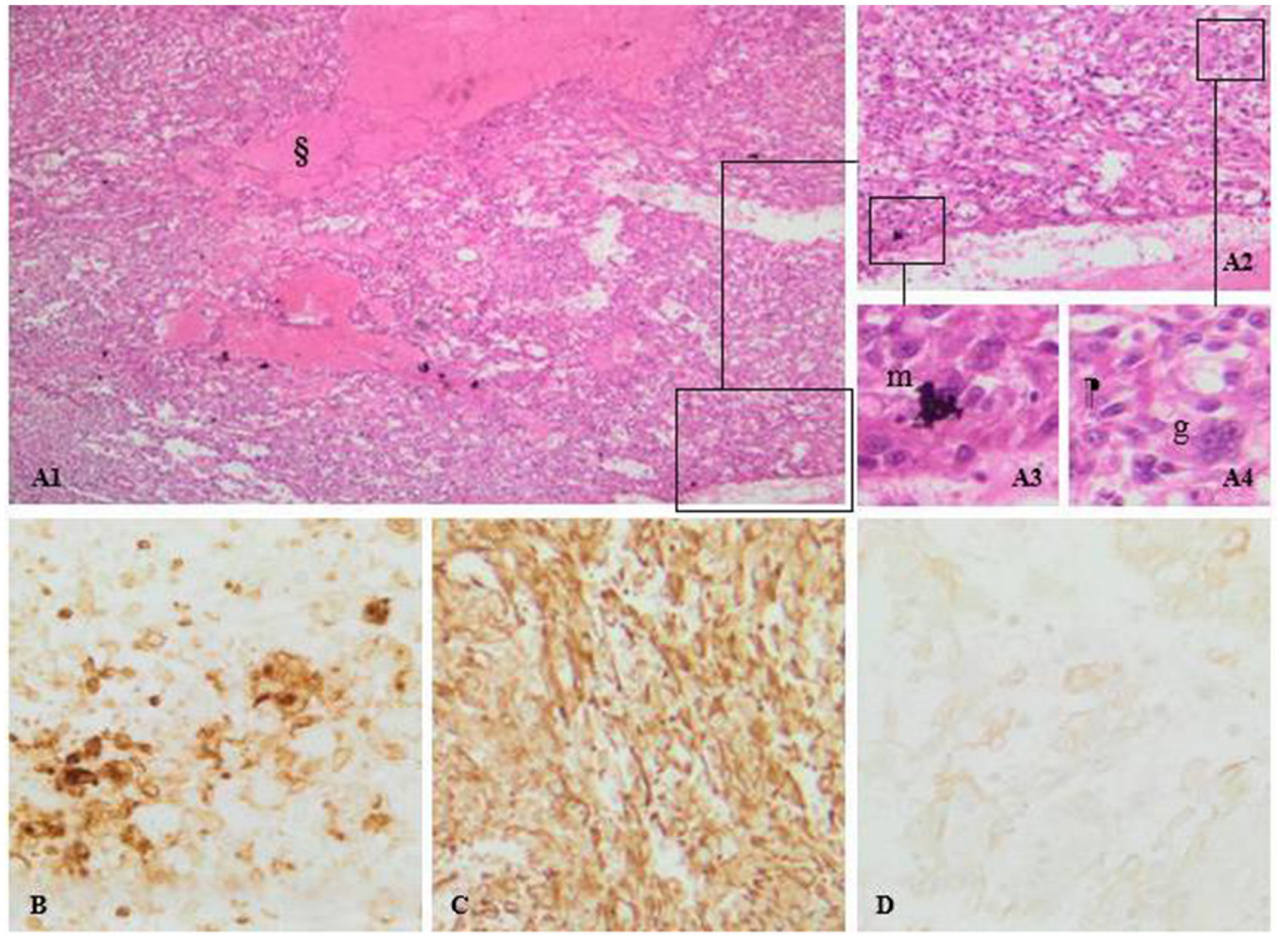
(**A**) Sarcomatoid renal cell carcinoma, PAX 8 positive, PDL1 negative. Pictures A1, A2, A3 and A4: H&E staining photomicrographs shows pleimorphic atypical spindle cells (m), along with multinucleated giant cells (g) arising from clear cell renal cell carcinoma. Note the mitotic activity and the area of necrosis (§). (**B**) CD10 expression confirms the clear cell type of the tumor. Diffuse vimentin stain positivity. (**C**) sarcomatoid differentiation of the tumor. (**D**) mild cytokeratin staining thus, an epithelial origin.

Pathology of the removed masses showed evidence of metastasis of sarcomatoid renal cell carcinoma, PAX 8 positive, PDL1 negative (Figure 4).

The case was discussed in the multidisciplinary uro-oncology team meeting at our institution and the decision was to reinitiate Sutent with close follow up.

**Figure 5:**
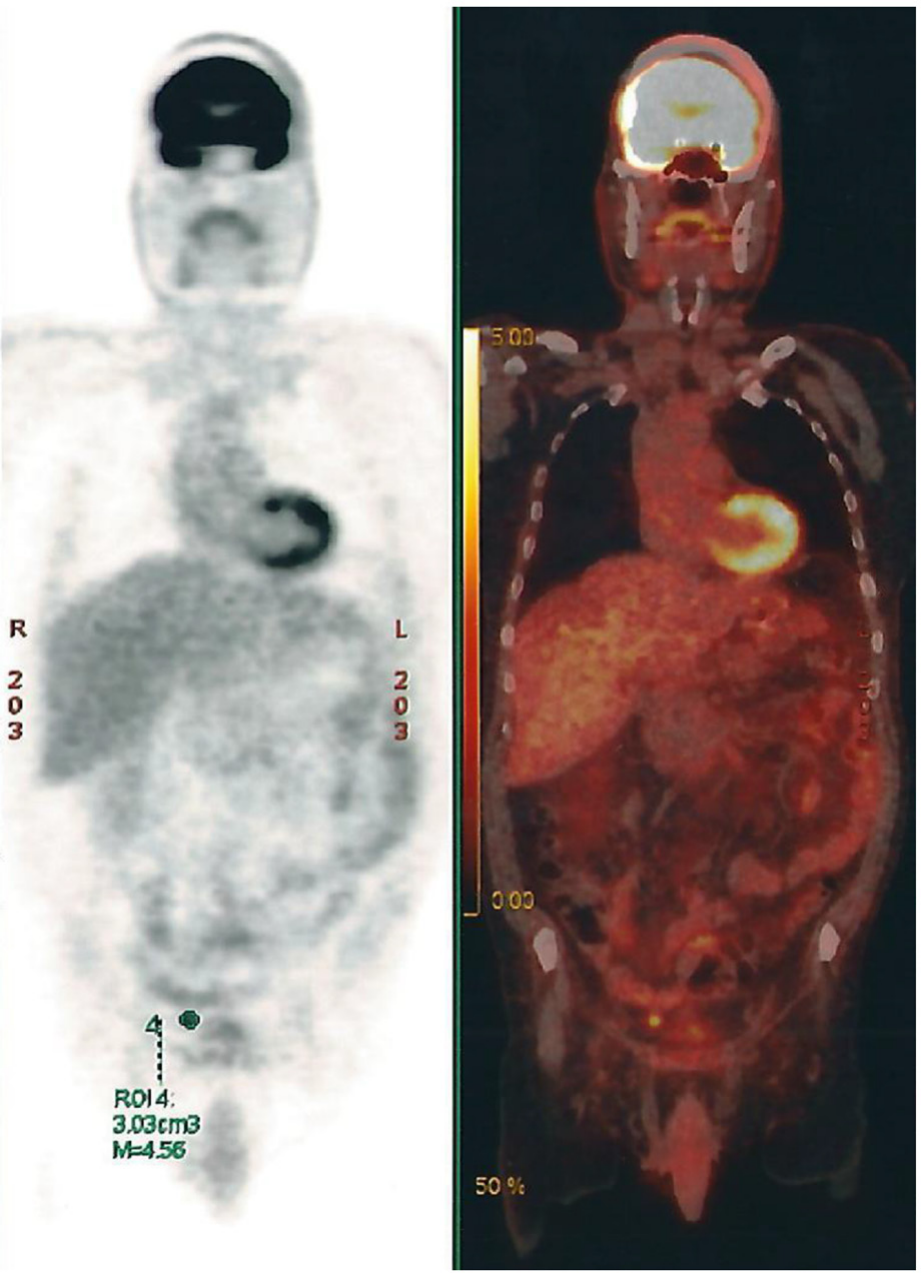
PET-CT Scan showing multiple retroperitoneal, pelvic and abdominal lymph nodes.

Four months later, a PET-CT scan was done and showed multiple retroperitoneal, pelvic and abdominal lymph nodes (Figure 5).

We did a second laparotomy and we found no evidence of peritoneal carcinosis; so we removed all the masses showed by the CT and performed a partial cystectomy due to the invasion of the bladder dome.

The pathology, once again, showed metastases of previously known RCC with sarcomatoid component.

Sunitinib was held and the patient started Cabozantinib 60 mg one tab per day, which was well tolerated.

**Figure 6:**
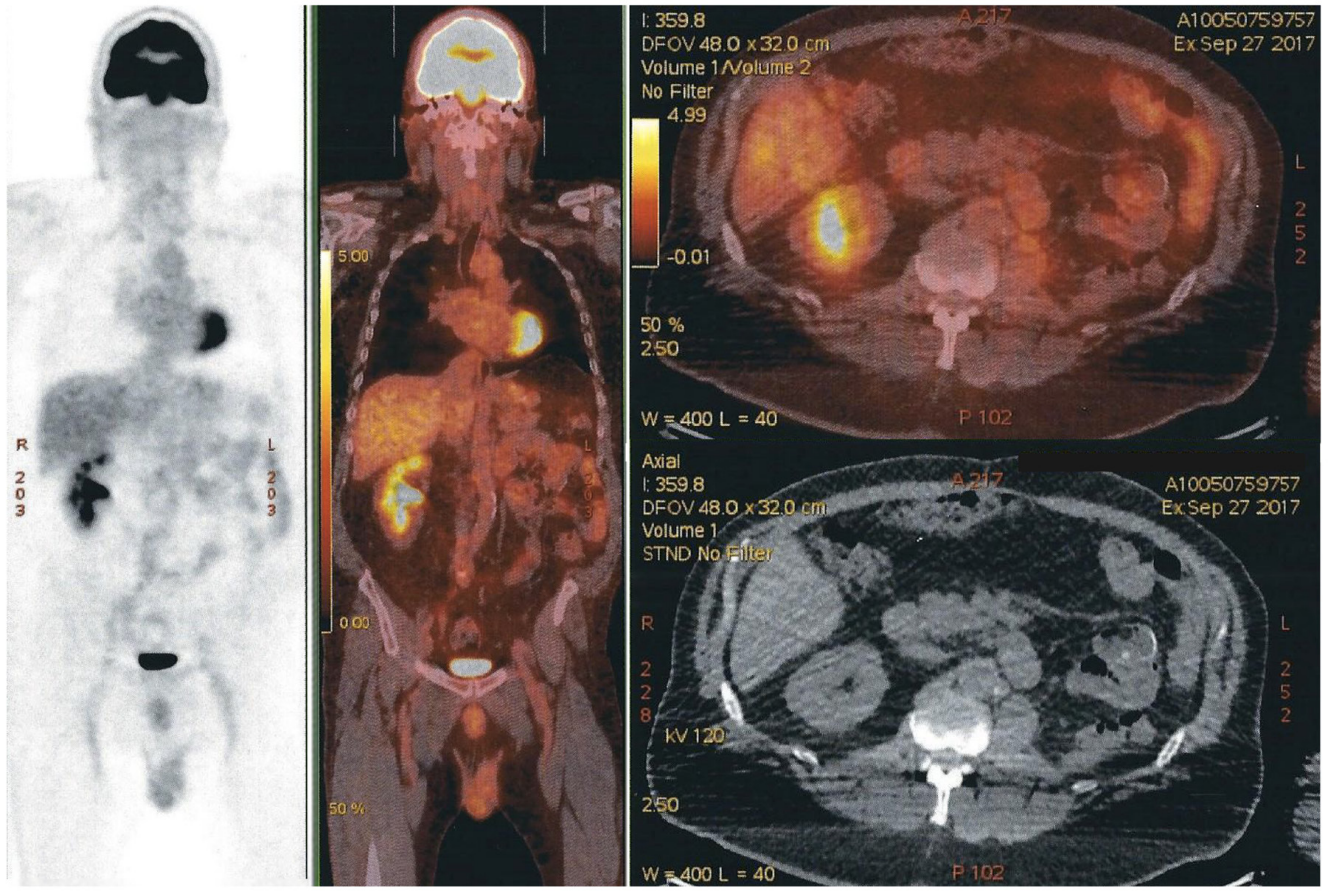
PET-CT scan with no evidence of recurrence of oncologic disease. Free left renal-bed, absence of distal lymph nodes, and nonexistence of any new suspect lesion. The modified area on the right side of the image is a shape added to cover the name of our patient.

PET-CT scans done 4 and 8 months later, showed no evidence of recurrence of oncologic disease (Figure 6).

Currently, our patient’s pet scan is negative and he is having a normal life, although the treatment was held 6 months ago, while he was judged to be on palliative care, three years ago.

## DISCUSSION

Sarcomatoid dedifferentiation occurs more frequently in CRCC (5–8% of cases) and Chromophobe RCC (8–9%) than in papillary RCC (3–4%) [3].

The sarcomatoid component presents as a pleomorphic spindle cell proliferation that merges with the carcinomatous component on histology [3].

Grading of the carcinomatous component of the tumor does not affect overall survival and the prognosis, but the presence of the sarcomatoid component is considered as Fuhrman nuclear grade 4 [3].

Invasion of adjacent organs is common, and median survival has been less than 1 year in most series [3].

Adibi et al., concluded that the percentage of sarcomatoid component is a prognostic factor in patients with sRCC, with larger percentage of involvement portending a worse survival, especially in patients with metastatic disease [4].

Takeuchi et al. demonstrated that the presence of low signal intensity areas on T2-weighted images corresponding to the area showing a hypovascular nature and markedly restricted diffusion might be characteristic findings of SRCC. Intratumoral hemorrhage and necrosis are present, but are not specific findings [5].

The genomic features underpinning renal cell carcinoma with sarcomatoid dedifferentiation are not well understood.

Sarcomatoid chromophobe RCC frequently have multiple gains (polysomy) of chromosomes 1, 2, 6, 10 and 17 [6].

Maalouf et al. demonstrated the presence of *TP53* mutations in sRCC and identified frequent mutations of neurofibromin 2 gene *(NF2)* that were mutually exclusive with *TP53* mutations [7].

On immunohistochemistry they are positive for MIB-1, AE1/AE3, vimentin, EMA that supports epithelial origin [8].

Other studies demonstrated the loss of immunostaining of the cell adhesion molecule cadherin-6 in sarcomatoid renal carcinoma [9].

Higher expression of programmed cell death ligand 1 (PD-L1) and programmed cell death 1 (PD-1), and CD8-positive cell density in sRCC compared to grade 4 ccRCC were demonstrated in a recent study conducted by Kawakami et al. The results indicate a high immunosuppressive characteristic of sRCC; PD-1/PD-L1 blockade may be a potential therapeutic option for sRCC [10]. In the case of our patient, PD-L1 was negative.

The treatment of metastatic renal cell carcinoma with sarcomatoid dedifferentiation remains a challenge with no well-defined strategies.

Thomas et al. proved by a matched controlled analysis that there is no statistically significant survival improvement in patients with SRCC treated by metastasectomy after radical nephrectomy [11].

No adjuvant therapies are actually recommended after nephrectomy for patients with renal cell carcinoma (RCC) [12]. Drugs that target the vascular endothelial growth factor receptor (VEGF-R) such as sunitinib and sorafenib are effective for disease control in the metastatic setting but they can rarely eradicate the disease [13]. In our case report, Sunitinib was not effective and recurrence of multiple intra and extra peritoneal masses and adenopathies was detected after 4 months of treatment.

On the other hand, Beuselinck et al. demonstrated that patients with metastatic CRCC, in whom the sarcomatoid component represent ≥ 25% of the total tumor volume, have a very poor response to anti-Vascular Endothelial Growth Factor Receptor Tyrosine Kinase Inhibitors [14].

Cabozantinib is a non-specific tyrosine kinase inhibitor with antineoplastic activity. It binds and inhibits multiple receptors such as VEGFR-1, VEGFR-2, VEGFR-3, MET (hepatocyte growth factor receptor), RET (rearranged during transfection), KIT (Mast/stem cell growth factor), FLT-3 (FMS-like tyrosine kinase 3), TIE-2 (TEK tyrosine kinase, endothelial), TRKB (tropomyosin-related kinase B) and AXL [15].

Cabozantinib is superior to everolimus in terms of PFS and OS in patients failing one or more lines of VEGF-targeted therapy [16]. So, most guidelines recommend offering Cabozantinib for ccRCC after one or two lines of vascular endothelial growth factor (VEGF)-targeted therapy in metastatic RCC.

In this case, the use of cabozantinib after Sunitinib failure was successful, but we think that it is the metastasectomy who had a benefic effect on disease free survival of our patient without any recurrence under cabozantinib.

To note, CARMENA trial concluded that there is no benefit from cytoreductive nephrectomy associated with sunitinib compared to the use of sunitinib alone in metastatic kidney cancer patients [17], but no data exists on the effect of metastasectomy before cabozantinib, this is why we think that this case allows us to discuss one more time, the indication of metastasectomy in the management of metastatic RCC.
